# Analysis of the optimum internal margin for respiratory‐gated radiotherapy using end‐exhalation phase assessments using a motion phantom

**DOI:** 10.1120/jacmp.v13i2.3715

**Published:** 2012-03-08

**Authors:** Yuji Yaegashi, Kunihiko Tateoka, Takuya Nakazawa, Kazunori Fujimoto, Katsumi Shima, Junji Suzuki, Akihiro Nakata, Yuichi Saito, Tadanori Abe, Koichi Sakata, Masato Hareyama

**Affiliations:** ^1^ Department of Radiation Oncology and Medical Physics Sapporo Medical University Graduate School of Medicine Sapporo Japan

**Keywords:** 4D CT, internal target volume, gated radiotherapy, internal margin, end‐exhalation

## Abstract

We aimed to optimize internal margin (IM) determination for respiratory‐gated radiotherapy using end‐expiratory phase assessments using a motion phantom. Four‐dimensional computed tomography (4D CT) data were acquired using a GE LightSpeed RT CT scanner, a respiratory‐gating system, and a motion phantom designed to move sinusoidally. To analyze the accuracy of 4D CT temporal resolution, a 25.4 mm diameter sphere was inserted into the motion phantom, and we measured the differences in sphere diameters between static and end‐exhalation phase images. In addition, the IM obtained from the maximum intensity projection within the gating window $\left({\text{MIP}}_{\text{GW}}\right)$ image was compared to theoretical value. Cranial–caudal motion displacement ranged from 5.0 to 30.0 mm, and the respiratory period ranged from 2.0 to 6.0 sec. Differences in sphere diameters between static and end‐exhalation phase images ranged from 0.37 to 4.6 mm, with 5.0 ‐mm and 30 mm target displacements, respectively. Differences between the IM obtained from the ${\text{MIP}}_{\text{GW}}$ and the theoretical values ranged from 1.12 to 6.23 mm with 5.0 mm and 30 mm target displacements, respectively. These differences increased in proportion to the target velocity due to a motion artifact generated during tube rotation. In this study, the IMs obtained using the ${\text{MIP}}_{\text{GW}}$ image were overestimated in all cases. We therefore propose that the internal target volume (ITV) for respiratory‐gated radiotherapy should be determined by adding the calculated value to the end‐exhalation phase image. We also demonstrate a methodology for subtracting motion artifacts from the ITV using a motion phantom.

PACS numbers: 87.53.Kn, 87.55.Gh, 87.56.jk

## I. INTRODUCTION

Stereotactic body radiotherapy (SBRT) is often used to treat early‐stage tumors in the lungs and abdomen.^(^ ^1^ ^–^ ^8^ ^)^ To achieve therapeutic goals, one of the most important considerations is to minimize normal tissue toxicity while maximizing a tumor absorbed dose. Therefore, the internal target volume (ITV) should be reduced as much as possible, taking into account respiration‐induced target displacement. The AAPM Task Group Report 76 recommends that if the target displacement is greater than 5 mm, a method to account for respiratory motion should be considered.^(9)^

Reduction of the treatment margin can be achieved by several methods, including breath holding,^(^ ^10^ ^,^ ^11^ ^)^ forced shallow breathing,^(12)^ and respiratory‐gated techniques.^(^ ^13^ ^–^ ^16^ ^)^ Respiratory‐gated radiotherapy incorporates an infrared tracking camera and a reflective marker placed on the patient's chest or abdomen. The respiration waveform generated by the marker motion is used to calculate the respiratory period for which a gating window can be set, to turn the radiation beam either on within the gate or off outside the gate. The gating window determines the selection of an image subset from a 4D CT dataset.

Most often, the gating window is selected near the end‐exhalation phase, when target displacement has the smallest variation and is more reproducible than during the end‐inhalation phase.^(^ ^17^ ^–^ ^19^ ^)^ The 4D CT dataset used in this study comprised 10 respiratory phases. The end‐inhalation phase was typically defined as the 0% phase and the end‐exhalation phase was defined as the 50% phase. In clinical practice, the end‐exhalation phase image was determined by the upward displacement of the diaphragm (cranial–caudal motion) from coronal and sagittal views using 4D CT.

**Figure 1:**
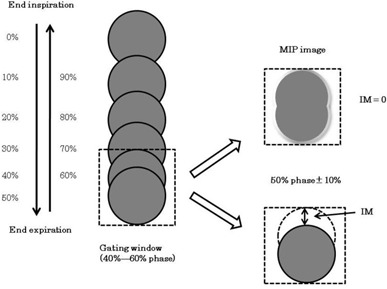
After the 4D CT images were scanned and reconstructed, all reconstructed images were sorted into 4D‐ CT images at 0%–90% phases, in increments of 10%. The 0%‐phase image typically defines the end‐inhalation image, and the end‐exhalation image is usually represented by the 50%‐phase image. The IM is determined either by the target displacement within the gating window (50% phase ±10%) or by using an MIP image (IM=0).

After 4D‐CT scanning, the acquired image dataset is transferred to a radiotherapy planning system for contouring and planning. The ITV is generated from a maximum intensity projection (MIP) image^(^ ^20^ ^–^ ^22^ ^)^ or by using only end‐exhalation phase image (50%‐phase image) within the gating window. Because the respiration‐induced target displacement is already included in the MIP image, it is not necessary to add an internal margin (IM) to the clinical target volume (CTV) (i.e., IM=0). In contrast, when the ITV is generated from the 50%‐phase image, the IM of the target displacement within the gating window should be added to the CTV (Fig. 1).

To select the gating window, Zhao et al.^(23)^ reported the results of motion analysis in 12 patients with lung tumors. They found that the IM could be defined by 4–7 phases around the 50% phase, when the target displacement was within 5 mm. However, we chose a gating window between the 40% and 60% (50%±10%) phases, as reported by Vedam et al.,^(24)^ to reduce the ITV as much possible. To evaluate the IM, we scanned and verified a respiratory motion phantom under various movement conditions using 4D CT. The purpose of this study was to optimize the IM determination for respiratory‐gated radiotherapy using only end‐exhalation phase image assessments from a motion phantom.

## II. MATERIALS AND METHODS

### A. Respiration‐correlated 4D CT data acquisition

Respiration‐correlated 4D CT data were acquired using a 4‐slice CT scanner in axial cine mode (LightSpeed RT, GE Medical Systems, Waukesha, WI). The cine images were acquired using a 0.7 sec scan time and a 2.5 mm slice thickness. The time interval between two consecutive CT image reconstructions at each position was 0.25 sec. During the scanning procedure, the respiratory waveform was recorded using a real‐time position management (RPM) gating system (Varian Medical Systems, Palo Alto, CA) by tracking the motion of infrared markers placed on the motion phantom. After 4D CT scanning, the Advantage 4D application (GE Medical Systems, Waukesha, WI) was used to retrospectively sort all the 4D CT images into 10 respiratory phases. To reduce the target volume, we selected a gating window within the phases at 50%±10%.

### B. Phantom study

**Figure 2:**
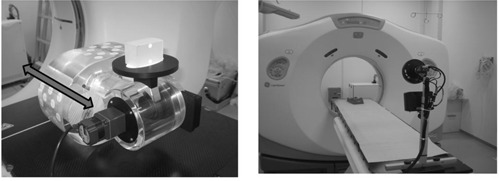
The respiratory motion phantom has sinusoidal movements. Target displacement occurs in the cranial–caudal direction (bidirectional arrow) with variable velocity and amplitude.

Target displacements were simulated using a respiratory motion phantom (QUASAR, Modus Medical Devices, London, ON) as shown in Fig. 2; the phantom moved sinusoidally. A sphere (diameter=25.4mm) was inserted into the motion phantom. To correspond with typical patient respiratory periods, the displacement of cranial‐caudal motion ranged from 5.0 mm to 30.0 mm, and the respiratory period ranged from 2.0 sec to 6.0 sec. After the sphere motion was scanned three times for each condition using 4D CT, the selected respiratory phase images were imported into a radiotherapy planning system (Eclipse 8.1; Varian Medical Systems, Palo Alto, CA). The sphere diameter was measured as described below.

### C. Limitations of temporal resolution during 4D CT

To investigate limitations of temporal resolution during 4D CT, the differences in sphere diameters between the static image and the 50%‐phase image were determined. The static image of the sphere was obtained using 4D CT with the motion phantom, which was set to zero amplitude. Due to the phantom's sinusoidal motion, the 50%‐phase image should have had almost no motion. Therefore, the sphere diameters in both the static and 50%‐phase image should be theoretically equal. All measurements were made with the same display scale.

### D. Accuracy of ITV from a MIP image

The MIP image was automatically generated using Advantage 4D from the 40%‐, 50%‐, and 60%‐phase images $\left({\text{MIP}}_{\text{GW}}\right)$. The IM on the ${\text{MIP}}_{\text{GW}}$ reflected the difference between the diameter of the sphere in the ${\text{MIP}}_{\text{GW}}$ image and that of the static sphere image. Here, the IM is the displacement from the end‐exhalation phase (i.e., the IM shown in Fig. 1).

The theoretical value of IM $\left({\text{IM}}_{\text{calc}}\right)$ was calculated from a sine function within the gating window. ${\text{IM}}_{\text{calc}}$ was calculated as follows:

(1) $$IMcalc=\left|\frac{D}{2}\cos\left(\frac{2\pi }{T}•0.5T\right)-\frac{D}{2}\cos\left(\frac{2\pi }{T}•pT\right)\right|$$

where *D* is the maximum target displacement during the respiratory period; D/2, the amplitude of the cosine function; *T*, the respiratory period; 2π/T, the angular velocity; 0.5, the percentage for the end‐exhalation phase (50%); and *p*, an arbitrary percentage for the gating window.

## III. RESULTS

**Figure 3:**
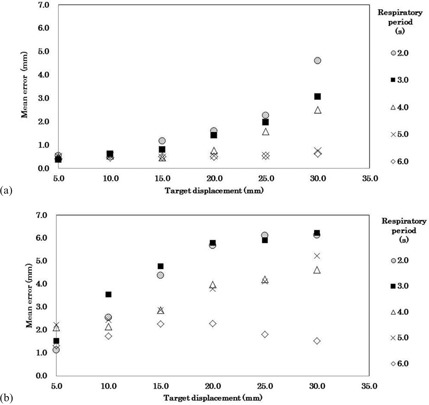
Differences between target displacement and the motion errors of the phase images within the gating window for each respiratory period: (a) comparisons of the diameter of a static sphere and a 50%‐phase image, (b) comparisons of MIP images (40%, 50%, 60% phases) to the theoretical values for target displacement.

The mean errors in sphere diameters between static‐phase images and 50%‐phase images ranged from 0.37±0.01mm (mean ±1 standard deviation) to 4.6±0.05mm (Fig. 3(a)) with 5.0 mm and 30.0 mm target displacements, respectively. Respiratory periods of 5 sec and 6 sec showed <1.0mm errors. However, for respiratory periods shorter than 4 sec, spheres had longer diameters than their actual diameters, and the errors increased in proportion to the target displacement.

The differences between the IM obtained from the ${\text{MIP}}_{\text{GW}}$ image and ${\text{IM}}_{\text{calc}}$ ranged from 1.12±0.01mm to 6.23±0.05mm with 5.0 mm and 30.0 mm target displacements, respectively (Fig. 3(b)).

## IV. DISCUSSION

### A. Relationship between errors and target velocity

**Figure 4:**
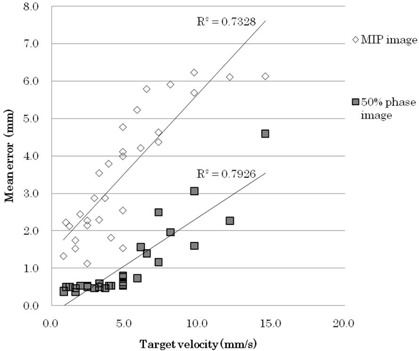
Correlations between motion errors and maximum target velocities within the 40%–60% gating window.

**Figure 5:**
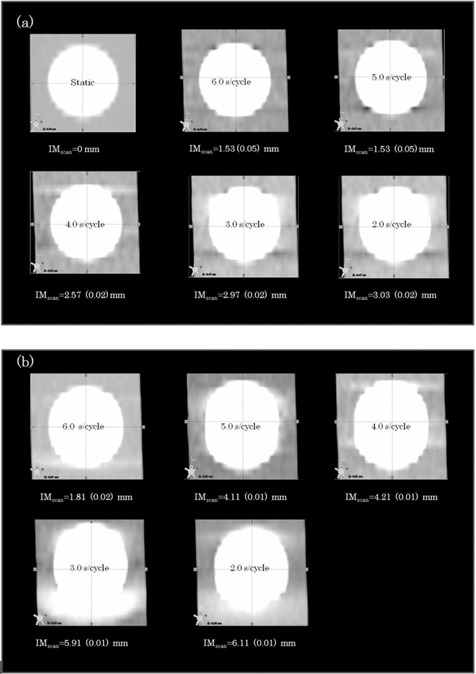
Example of a sphere image at a respiratory amplitude of 25.0 mm: (a) a 50%‐phase image, and (b) MIP images (40%, 50%, 60% phases) for each respiratory period. Motion artifacts appeared as ${\text{IM}}_{\text{scan}}$ with increasing respiratory period. The numbers in parentheses are the standard deviations.

Figure 4 shows the relationship between the errors and the maximum target velocity for each condition during the end‐exhalation phase. The errors increased with increasing target velocity. Figures 5(a) and 5(b) show that motion artifacts increased with shorter respiratory periods. The motion artifact of the ${\text{MIP}}_{\text{GW}}$ image (Fig. 5(b)) was larger than that of the 50%‐phase image (Fig. 5(a)). Thus, the errors resulted from motion artifacts, because the 4D CT images included target displacement during the 0.7 sec tube rotation.

In this study, we defined the target displacement during tube rotation as ${\text{IM}}_{\text{scan}}$. ${\text{IM}}_{\text{scan}}$ is useful only if the motion artifact is measured using the motion phantom with the specific 4D CT scanner to be used at each institution. Furthermore, since ${\text{IM}}_{\text{scan}}$ depends on the tube rotation time, its value differs with scanning time.

### B. Estimation of an MIPGW image using 4D ‐CT

Several studies have shown that the ITV can be determined from the ${\text{MIP}}_{\text{GW}}$ for respiratory‐gated radiotherapy using 4D CT.^(^ ^20^ ^–^ ^23^ ^)^ However, in this study, the ITV obtained using the ${\text{MIP}}_{\text{GW}}$ image was overestimated in all cases, as shown in Fig. 4. Because the ${\text{MIP}}_{\text{GW}}$ image includes some midexhalation‐phase images with fast target velocities, it is strongly affected by the motion artifact. Several studies have demonstrated that the ITV can be underestimated due to the influence of breathing irregularities; this occurred when the ${\text{MIP}}_{\text{GW}}$ image was generated using all the respiratory phases or because the gating window was selected widely.^(^ ^25^ ^–^ ^27^ ^)^ However, the ${\text{MIP}}_{\text{GW}}$ was overestimated when the ${\text{MIP}}_{\text{GW}}$ was generated using only phases near the end‐exhalation phase, just as in our results. Therefore, we propose that the ITV for respiratory‐gated radiotherapy should be determined by adding ${\text{IM}}_{\text{calc}}$ to the 50%‐phase image, rather than by using the ${\text{MIP}}_{\text{GW}}$ image. However, for a target with respiratory motion, the ITV generated from the MIP of 4D CT is clearly more accurate than what can be obtained using the “slow CT scanning technique.”

### C. IM optimization for respiratory‐gated radiotherapy using end‐exhalation phase images

The ITV will be overestimated if ${\text{IM}}_{\text{scan}}$ is not subtracted from ${\text{IM}}_{\text{calc}}$ when the target velocity is extremely high. We thus propose a methodology for determining the IM within the gating window $\left({\text{IM}}_{\text{GW}}\right)$ by using the 50%‐phase image as follows:

(2) $$IM_{GW}=IM_{calc}-IM_{scan}$$

(3) $$=\left|\frac{D}{2}\cos\left(\frac{2\pi }{T}•0.5T\right)-\frac{D}{2}\cos\left(\frac{2\pi }{T}•pT\right)\right|-IM_{scan}$$

**Figure 6:**
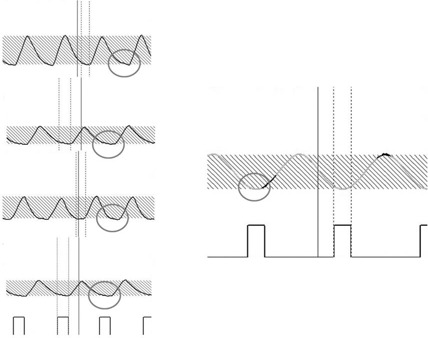
Comparison of a typical patient's respiratory waveform (left) and the motion phantom with a sine curve (right). The patient's respiratory waveform is more static than the sine curve near the end‐exhalation phase (circles).

Although ${\text{IM}}_{\text{calc}}$ is based on sinusoidal motion, we believe that clinical application is feasible for respiratory‐gated radiotherapy. The static state at the end‐exhalation phase of a typical patient's respiratory waveform is longer than the sinusoidal curve obtained when using a motion phantom (Fig. 6). Therefore, if ${\text{IM}}_{\text{calc}}$ is used for respiratory‐gated radiotherapy, the generated ITV includes the patient's actual target displacements. However, as our results indicate, ${\text{IM}}_{\text{GW}}$ should be taken into account when a patient's respiratory period is short (<3sec) and has a large target displacement (>2cm).

### D. Accuracy of image reconstruction at the end‐exhalation phase

**Figure 7:**
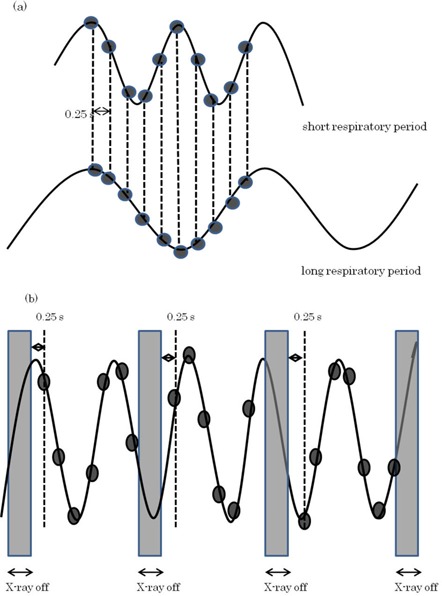
Accuracy of 4D CT image reconstruction at the end‐exhalation phase (dots indicate the locations of the image reconstruction): (a) the number of image reconstructions increases or decreases depending on the respiratory period, (b) the location of the image reconstruction shifts in the X‐ray off period during CT couch relocation.

When the IM was generated from end‐exhalation phase data, image reconstruction may not have been accomplished exactly at the 50%‐phase location for the following reasons. First, because the image reconstruction interval is constant, the image dataset decreases with a shorter respiratory period, and image reconstruction may not necessarily be accomplished by the end‐exhalation phase as it is with the 50% phase (Fig. 7(a)).

Second, as illustrated in Fig. 7(b), the starting point for data acquisition at each position shifted slightly. This was because the X‐ray was turned off while the table was moved to the next position during 4D‐CT.^(21)^ Table 1 shows the variations in positions that occurred at the 50% phase, using the motion phantom as a check on the influence of these sources of error. The mean phase position of the end‐exhalation phase image was 49.6%±1.2%, and the target displacement variation was less than 0.07 mm. Therefore, the 50%‐phase image was accurately reconstructed at the end‐exhalation phase location used in this study. For clinical use, however, it will be necessary to check that the deviation at the end‐exhalation phase is within tolerable limits for each patient. When a patient's respiration deviates from the normal pattern, Varian's RPM gating system with its predictive filter automatically turns the beam off. However, if a patient's breathing is extremely irregular during a 4D CT scan, we recommend switching to conventional radiotherapy.

**Table 1:** Variations in position of the 50% phase using a sinusoidal motion phantom during 4D CT scans.

*Amplitude (mm)*	*Breathing Period (sec)*	*Phase Range (%)*	*Mean (%)*	*SD (%)*
	6.0	48–51	49.8	1.23
	5.0	48–52	49.2	1.40
30.0	4.0	47–52	49.3	1.89
	3.0	47–52	50.6	1.51
	2.0	46–54	52.7	2.67
	6.0	48–51	49.9	0.83
	5.0	48–51	50.0	1.07
25.0	4.0	47–51	49.6	1.60
	3.0	47–53	50.4	2.88
	2.0	46–54	47.9	3.04
	6.0	48–51	49.3	0.89
	5.0	47–52	50.8	1.91
20.0	4.0	48–51	50.0	1.07
	3.0	45–49	48.3	1.39
	2.0	48–55	50.0	2.27
	6.0	49–52	50.3	1.04
	5.0	47–52	50.8	1.91
15.0	4.0	49–52	51.1	1.13
	3.0	45–53	48.9	2.36
	2.0	44–52	47.5	2.62
	6.0	48–51	49.5	1.41
	5.0	48–52	49.8	1.49
10.0	4.0	47–53	49.5	2.20
	3.0	45–52	48.8	2.43
	2.0	44–55	49.1	4.97
	6.0	48–51	49.5	1.22
	5.0	48–52	49.8	1.72
5.0	4.0	47–52	50.0	2.45
	3.0	47–53	51.0	2.53
	2.0	44–51	46.0	2.90

Note that the two above‐mentioned reasons pertain only to cine 4D CT, and this issue has not been studied for helical 4D CT.

To use the methodology described in this study properly, it is very important to employ immobilization devices (stereotactic body frames, vacuum cushions, etc.), which serve to immobilize the patient and provide accurate tumor localization. Negoro et al.^(12)^ reported that tumor displacement was significantly reduced, from a range of 8–20mm to a range of 2–11mm, by the use of immobilization devices.

Future studies will further improve the accuracy of respiratory‐gated radiotherapy by adding retrospective analysis of an electronic portal imaging device in the cine mode^(^ ^28^ ^,^ ^29^ ^)^ and audiovisual biofeedback^(30)^ to the method using ${\text{IM}}_{\text{GW}}$.

## V. CONCLUSIONS

We have demonstrated a method for optimizing the IM for respiratory‐gated radiotherapy using end‐exhalation phase image assessments using the motion phantom. When the ITV was generated using the ${\text{MIP}}_{\text{GW}}$ image, it was overestimated in all cases. We therefore recommend that the ITV for respiratory‐gated radiotherapy should be determined by adding ${\text{IM}}_{\text{calc}}$ to the 50%‐phase image. Moreover, in order to reduce the ITV, we propose that the motion artifact generated during tube rotation $\left({\text{IM}}_{\text{scan}}\right)$ should be subtracted from ${\text{IM}}_{\text{calc}}$ when a patient's respiratory period is relatively short (<3sec) and the degree of target displacement is high (>2cm).

## ACKNOWLEDGMENTS

The authors wish to thank Prof. Jun Takada and Dr. Kenichi Tanaka of the Radiation Protection Laboratory (Sapporo Medical University) and Dr. Kenji Kagei of the Kushiro City General Hospital.
